# The Multiplicity of Infection of Recombinant Vaccinia Virus Expressing the T7 RNA Polymerase Determines the Rescue Efficiency of Vesicular Stomatitis Virus

**DOI:** 10.3389/fmicb.2022.846426

**Published:** 2022-04-04

**Authors:** Fan Yang, Jinlong Tan, Yongxiang Fang, Guohua Chen, Yongzhi Zhang, Qianqian Hu, Wuweiyi Han, Yongsheng Liu, Baoquan Fu, Zhizhong Jing, Weike Li

**Affiliations:** ^1^State Key Laboratory of Veterinary Etiological Biology, Key Laboratory of Veterinary Public Health of Ministry of Agriculture, Lanzhou Veterinary Research Institute, Chinese Academy of Agricultural Sciences, Lanzhou, China; ^2^College of Animal Science, Anhui Science and Technology University, Fengyang, China; ^3^Hebei Key Laboratory of Preventive Veterinary Medicine, College of Animal Science and Technology, Hebei Normal University of Science and Technology, Qinhuangdao, China; ^4^State Key Laboratory of Veterinary Etiological Biology, Key Laboratory of Veterinary Parasitology of Gansu Province, Lanzhou Veterinary Research Institute, Chinese Academy of Agricultural Sciences, Lanzhou, China; ^5^Jiangsu Co-innovation Center for Prevention and Control of Important Animal Infectious Diseases and Zoonoses, Yangzhou, China

**Keywords:** vesicular stomatitis virus, vTF-7.3, reverse genetic, rescue efficiency, the multiplicity of infection

## Abstract

Vesicular stomatitis virus (VSV) has a wide range of cell tropism, making it a prototype of studying the negative-strand RNA virus (NSRV), including virus–host interactions and vaccine development. Although VSV rescue systems have been progressively optimized throughout time, the T7-based expression system is the most commonly utilized to rescue VSV. However, it remains a significant barrier for many labs. In our study, we found that rescue VSV’s efficiency is associated with the various multiplicities of infection (MOIs) of recombinant vaccinia virus expressing the T7 RNA polymerase (vTF-7.3). It works at maximum efficiency while the MOI of vTF-7.3 is 5, which is analyzed by quantitative PCR, Western blot, and flow cytometry, compared to the lowest rescue level with MOI of 1. Meanwhile, our data also suggest that purification of vTF-7.3 prior to transfection is a prerequisite for VSV rescue. Overall, our study reveals for the first time a precise correlation between vTF-7.3 and rescue efficiency, which may aid in resolving the uncertainties in the quest to build the VSV reverse genetic system.

## Introduction

Vesicular stomatitis virus (VSV) is an envelope with bullet-shaped, non-segmented negative-strand RNA virus (NSRV). It belongs to the *Rhabdoviridae* family, including the rabies virus (RV). Unlike RV infections, human VSV infections are typically subclinical or cause a mild flu-like illness or may go unnoticed since they are completely asymptomatic (Hanson et al., 1950; Patterson et al., 1958; Quiroz et al., 1988). The relative safety of the VSV combined with its abundant replication in a broad range of cultured cells has favored the use of VSV as a prototype tool to understanding NSRV (for example, Ebola virus, avian influenza), including fundamental host cell processes, molecular details of viral gene expression, the pathogenesis of viral infection, and the human vaccine vector (Filone et al., 2013; Wang et al., 2015; Ruedas and Connor, 2017; Heinrich et al., 2018; Case et al., 2020).

The advent of reverse genetic technology has revolutionized the field of RNA viruses, and along with the development of VSV rescue systems, vaccina virus-free rescue systems have also been developed (Garbutt et al., 2004). However, stable T7 cell lines or CMV promoters have significantly lower expression levels than the vaccinia-mediated system, commonly used in reverse genetic systems to rescue VSV (Buchholz et al., 1999; Harty et al., 2001; Inoue et al., 2003; Garbutt et al., 2004; Popov et al., 2009).

Almost all of the reports used to be infected with vTF-7.3 at an MOI of 5–20 before co-transfection (Whitt et al., 1989; Lawson et al., 1995; Ostertag et al., 2007; Tani et al., 2011; Li et al., 2018). However, no report has tried to optimize the condition for the vTF-7.3 infection, especially with the rescue efficiency and cell death (apoptosis), both important elements in reverse genetics.

Our study aimed to clarify the impacts of vTF-7.3 accurate measure, and it may be useful in resolving ambiguity during the construction of the VSV reverse genetic system.

## Materials and Methods

### Cell Lines and Viruses

BSR-T7 (a gift from Dr. Zhigao Bu, Harbin Veterinary Research Institute, CAAS) and BHK-21 cells were grown in Dulbecco’s modified Eagle’s medium (DMEM) (Gibco, Invitrogen, Carlsbad, CA, United States) high glucose supplemented with 5% FBS at 37°C with 5% CO_2_. CV-1 cells were grown in Roswell Park Memorial Institute (RPMI) 1640 medium supplemented with 10% FBS to develop recombinant vaccinia virus vTF-7.3 (kindly provided by Prof. Xianzhu Xia, Institute of Military Veterinary, Academy of Military Medical Sciences).

### Plasmid Construction

The wt full-length VSV (Indiana serotype) genome plasmid and helper plasmids P^N^, P^P^, and P^L^ were obtained from Dr. Zhigao Bu. The ORF of the mCherry red fluorescent protein gene was cloned and fused to the N-terminus of the P gene in the wt full-length VSV plasmid (VSVmc-p) using the overlap PCR (Figure 6), and the primers are described in Table 1.

**TABLE 1:** Primers used to insert the mCherry gene into the full-length genome of VSV.

Primer	Nucleotide sequence (5′–3′)
N(+)-F	CAGCCTGATGACATTGAGTATACATCT CTTACTACAGCAGG
N-mC(−)-R	TCCTCGCCCTTGCTCACCAT*GGTGGCGGC*GATA TCTGTTAGTTTTTTT
P(+)-F	ATGGACGAGCTGTACAAGggaggaaacagcggaggaATGGATA ATCTCACAAAAGTTCG
P-M(−)-R	GCCTATTAAGTCATTATGCCAATTTAAATCT GAGCTTGACGGGC
mC(+)-F	TATGAAAAAAACTAACAGATATC*GCCGCCACC*ATGGT GAGCAAGGGCGAGG
mC-P(−)-R	ACTTTTGTGAGATTATCCATtcctccgctgtttcctccCT TGTACAGCTCGTCCAT

*The underlined regions as the recognition site, BstZ17 I and Swa I, respectively. The Kozak sequence is italicized. Lowercase is the linker (GGNSGG) (Jiang et al., 2010; Li et al., 2018).*

### Propagation and Purification of Recombinant Vaccinia Cirus (vTF-7.3)

CV-1 cells were seeded to approximately 95–100% confluence around twenty 150-mm TC-treated culture dishes and infected with vTF-7.3 at MOI of 1. When more than 90% cytopathic effects (CPE) were observed, the media were removed and the infected cells harvested into 2–50 ml 10 mM Tris–HCl pH 8.8 buffer. The cells were centrifuged and resuspended twice, then frozen and thawed three times before being sonicated in a sonic bath for 2 min. 8 ml 36% sucrose was purified in buffer 10 mM Tris–HCl pH 8.8 as a cushion, leaving 22 ml for the sample. The pelleted sample was resuspended at the bottom in 10 mM Tris–HCl pH 8.8 and stored at −80°C after being centrifuged at 3,600 × *g* for 1.5 h at 4°C.

### Recovery of Recombinant Vesicular Stomatitis Virus

Recombinant VSVs (rVSVmc-p) were rescued using the previously described procedures (Lawson et al., 1995; Whelan et al., 1995; Altomonte et al., 2009; Whitt, 2010; Li et al., 2018). BSR-T7 cells on 60-mm dishes were infected at MOIs of 0.5, 1, 5, 10, and 20 with vTF-7.3, respectively. After 1 h, the full-length VSV genome plasmid encoding the mCherry gene (4 μg) and the N (6 μg), P (4 μg), and L (2 μg) plasmids were co-transfected into the cells by using Lipofectamine 2000 (20 μl). After a 48-h incubation at 37°C in 5% CO_2_, the culture media were collected and kept at −80°C.

### Fluorescence-Based Plaque Assay

Titration of the recovered virus was performed using BHK-21 cells. Briefly, cells were grown in 12-well plates to approximately 90% confluency and infected with the recovered virus 10-fold serially diluted in DMEM. After 1 h of incubation, the inoculum was removed and rinsed with 1 × phosphate-buffered saline (PBS) before being covered with 0.7 ml of 0.8% (wt/vol) agarose in DMEM. At 24 h after infection, the plaques were then examined for mCherry signal under fluorescence microscopy.

### Flow Cytometry Analysis

Flow cytometry was used to determine the proportion of rVSVmc-p-infected cells. Briefly, BHK-21 cells were infected with 200 μl rVSVmc-p from −80°C stock, with gentle rocking 1 h to allow the viruses to bind the cell. Cells were washed twice with PBS and analyzed by flow cytometry (BD Biosciences, San Jose, CA, United States). Each data point represents at least 5,000 events and three biological replicates.

### RNA Extraction and Quantitative Real-Time PCR

According to the manufacturer’s instructions, total cellular RNA was extracted using TRIzol (Invitrogen) and used in reverse transcription to generate cDNA as described previously (Rasmussen et al., 2003; Harouaka and Wertz, 2012). Quantitative real-time PCR (qPCR) was conducted using a PowerUp SYBR Green Master Mix according to the manufacturer’s instructions. All data were calculated using a 2^–ΔΔCT^ method as previously described (Livak and Schmittgen, 2001). Samples were normalized to the quantity of the β-actin gene. All of the primers used to measure VSV N and β-actin genes are shown in Table 2.

**TABLE 2:** Primers used for measuring the N gene of VSV and β-actin.

Primer	Nucleotide sequence (5′–3′)
N-F	CCGACAGCCTGATGACATTGAGT
N-R	CCATTCGACCACATCTCTGCCTTG
β-Actin-F	CTGTGCTATGTTGCCCTGGACTTC
β-Actin-R	CCGCTCGTTGCCAATGGTGAT

### Western Blotting

Western blots were prepared as described previously (Ryder et al., 2015). Briefly, cells were washed twice with cold PBS and lysed on ice for 30 min with RIPA lysis buffer (Solarbio, Beijing, China) supplemented with protease inhibitors. Samples were subjected to 8.5% Bis–Tris polyacrylamide gels and transferred to polyvinylidene fluoride membranes (Millipore, Bedford, MA, United States). Western blots were probed with specific antibodies for anti-VSV G and detected using the enhanced chemiluminescence detection kit (#1705062, Bio-Rad).

### Cell Viability Assay

According to the manufacturer’s instructions, the viability was determined using the CellTiter-Glo Luminescent Cell Viability Assay Kit (MTT) (Promega, Madison, WI, United States). Briefly, BHK-21 cells were infected with vTF-7.3 for 1 h, and then the medium was removed. Subsequently, 100 μl of reagent was added. Immediately after a 10-min incubation, the samples were measured in an Orion II Microplate Luminometer (Titertek-Berthold, Pforzheim, Germany).

### Statistical Analysis

The significance of differences between groups was determined using GraphPad Prism 7’s Student’s *t*-test. It was considered significant if the unadjusted *p*-value was less than 0.05.

## Results

### Non-purified Recombinant Vaccinia Virus Is Highly Toxic to Cultured Cells

**FIGURE 1:**
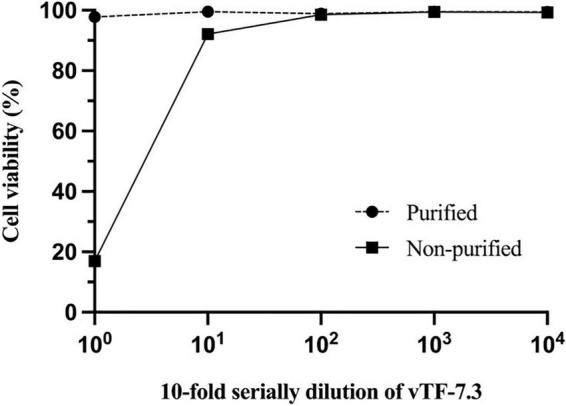
Cell cytotoxicity studies. Monolayers of BSR-T7 cells were incubated with 10-fold serial dilution of vTF-7.3 for 1 h., respectively. 1 × DPBS was used as the solvent control. MTT assays determined cell viability. Experiments were carried out three times, and error bars reflect the standard deviations.

To generate vTF-7.3, the cells need to be lysed to release virus particles since the vaccinia virus is unique among most DNA viruses in that its replication occurs in the cytoplasm of the infected host cells (Tolonen et al., 2001). However, cell lysis releases many metabolites, which are toxic to cells, such as histamine. Toxicity inhibits the growth of BRS-T7 cells, resulting in that the virus rescue is usually unsuccessful. Therefore, purified vTF-7.3 and non-purified vTF-7.3 were evaluated for cytotoxicity by MTT assays [3-(4,5-dimethyl-2-thiazolyl)-2,5-diphenyl-2H-tetrazolium bromide] using CellTiter 96 from Promega (Li et al., 2018). As shown in Figure 1, the toxicity of non-purified vTF-7.3 reduced until it reached 10^–2^ dilution, but the viral load also significantly decreased. In contrast, the purified vTF-7.3 showed almost little cytotoxicity.

### Comparison of Rescuing Vesicular Stomatitis Virus by Varying Multiplicities of Infections of vTF-7.3

The purified vTF-7.3 was added to the BSR-T7 cells prior to transfection by MOIs of 0.5, 1, 5, 10, and 20. Every group was repeated three times. After 40 h post-transfection, the mCherry fluorescence of one well was first observed in the group with MOI of 5, and another well in the same group also observed the fluorescence in the following 10 h. One well in the group with an MOI of one observed the fluorescence after 55 h post-transfection. No fluorescence was observed for the groups with MOI of 0.5, 10, and 20.

### Evaluation of Rescue Efficiency

To evaluate which MOI (0.5, 1, 5, 10, and 20) was the ideal condition for rescuing the virus, we measured it using several methods to ensure high accuracy and stability. This section is divided into the following subsections: fluorescence-based plaque assay, flow cytometry analysis, quantitative real-time PCR, and Western blot. All of the experiments were driven by the same stock at −80°C.

**FIGURE 2:**
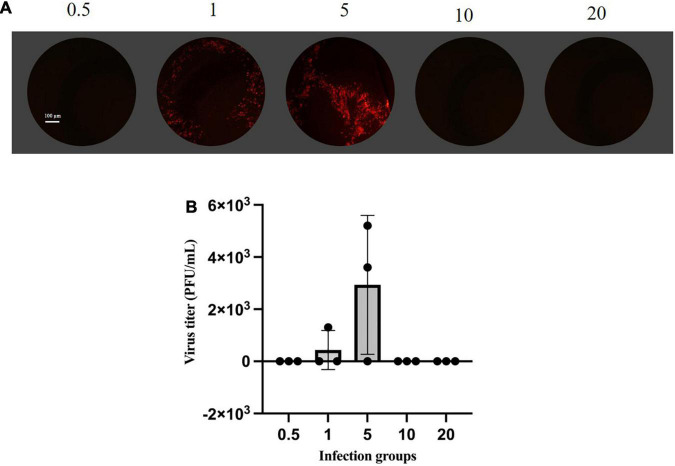
Plaque assay based on mCherry fluorescence. **(A)** Monolayers of BHK-21 cells were infected with rVSVmc-p for 24–48 h. Cells were then fixed, observed, and determined by plaque assay under fluorescence microscopy. **(B)** Titrations of rVSVmc-p. BHK-21 cells were incubated with different groups of MOIs. The virus titers were determined by titration on BHK-21 cells.

**FIGURE 3:**
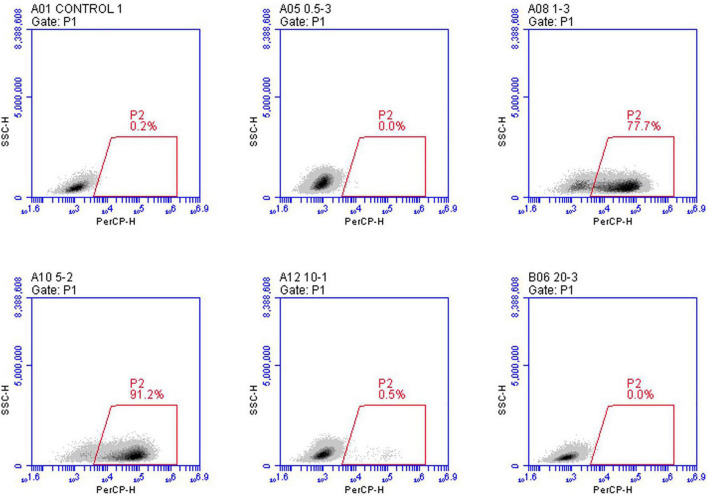
*In vitro* analysis of the infection efficiency under the mCherry channel using flow cytometry. rVSVmc-p was used to infect BHK-21 cells at the indicated MOIs three times. Infected cells have been examined using flow cytometry. mCherry fluorescence signal was used to determine infection potency.

**FIGURE 4:**
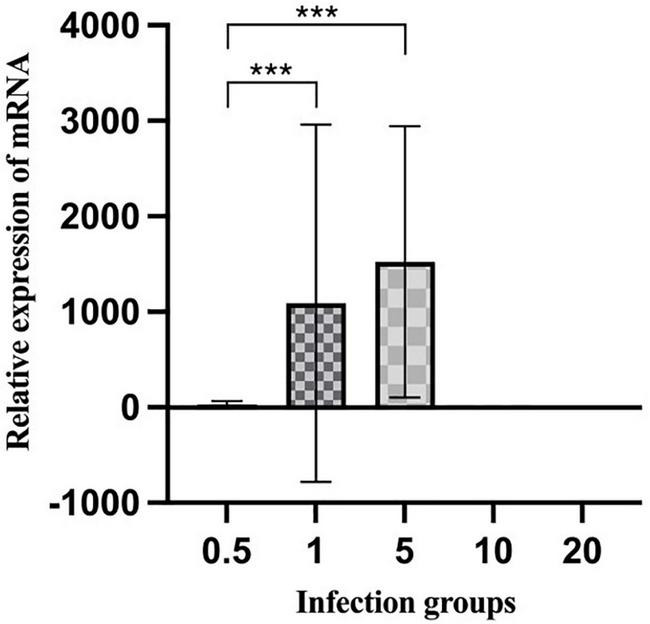
Expression levels of mRNA measured by qPCR. The total RNAs were extracted from the BHK-21 cells infected with different groups of rVSVmc-p and used in reverse transcription to generate cDNA. All data points were derived from at least four biological replicates, with the standard deviation shown by the error bars. *p*-values were calculated using a two-tailed *t*-test; *** indicates *p* < 0.01.

**FIGURE 5:**
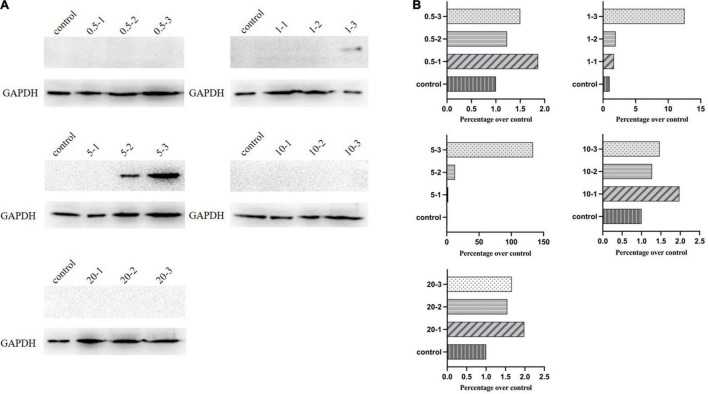
Western blot analysis of G protein expression. **(A)** Protein expression levels of G for the initial infection by Western blotting. **(B)** ImageJ measured the expression levels.

(1) **Fluorescence-based plaque assay**. The efficiencies of rescue were quantified by plaque-formation assay (van Remmerden et al., 2012; Ye et al., 2021). The plaque assay is the gold standard for determining the infectious titer of VSV. The fluorescence-based plaque assay is functional, stable, efficient, and direct compared to the traditional plaque assay. BHK-21 cells were infected with the supernatant cell cultures from rescue viruses. mCherry expression and plaque visualization only 24 h after assay setup are shown in Figure 2A, which resulted in clear and distinct fluorescent virus plaque formation. Analysis of mCherry expression showed that plaques from the supernatant of MOI of five infected cells were the highest titer and up to 5.2 × 10^3^ PFU/ml (Figure 2B).

(2) **Flow cytometry analysis**. Flow cytometry was utilized to assess infection efficiency quantitatively. We created a recombinant VSV expression of the mCherry fluorescent protein to do this. Flow cytometry analyses measured the percentage of mCherry + cells performed 24 h post-infection. The cells from three wells were harvested and analyzed with a flow cytometry instrument. In each case, the amounts of viral particles were counted with mCherry, validating the fluorescence data. As predicted, based on the calculated red particle ratio at each group, the results demonstrated that the group with the MOI of five had the highest cell ratio, with between 89.3 and 91.2% of the cells expressing mCherry fluorescent protein (Figure 3).

(3) **Quantitative polymerase chain reaction**. We further compared viral load using the C_*q*_ value, with low C_*q*_ values resulting from large amounts of the amplified product indicating higher viral loads (Ryabov et al., 2014; Bradford et al., 2017). SYBR Green measured samples from five groups for the detection and calculation of the β-actin housekeeping gene and the VSV N gene. As shown in Figure 4, BHK-21 cells infected at an MOI of five were significantly higher than group MOI of 1, indicating a higher proportion of infectious particles. This result is also in line with the flow cytometry analysis.

(4) **Western blotting for analysis of discrepant results**. We correlated these results with the flow cytometry and qPCR observations based on Western blot analysis. Protein expression was confirmed by using an anti-VSV G antibody. As already observed (Figure 5), consistent with low expression levels analyzed by flow cytometry and qPCR, the group with MOI of one showed a dramatic decrease, whereas MOI of five had the highest expression.

**FIGURE 6:**
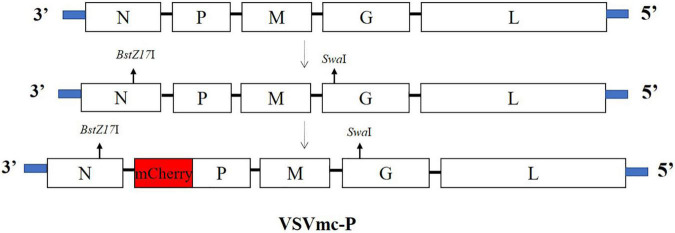
Schematic diagram of recombinant VSV expressing the mCherry gene (rVSVmc-p). The VSV genome is depicted in schematic representations (top), with insertion locations indicated by arrows (second) and VSV vectors including a transcription cassette encoding mCherry (bottom), respectively.

## Discussion

The advent of the reverse genetic system has made it possible to examine the function analysis of much of the viral genome in detail. As a prototype for researching NSRV, studies with VSV have resulted in the understanding of fundamental host cells processes, molecular pathogenesis of viral infection, and live recombinant vaccine vectors (Roberts et al., 1998; Liang et al., 2015; Marzi et al., 2019).

In the past 30 years, reverse genetics of VSV has been developed, including the use of stable cell lines, a plasmid expressing T7 polymerase, and the cellular pol II (CMV) promoter (Harty et al., 2001; Brown et al., 2011), as well as adding M- and G-proteins as helper plasmids (Garbutt et al., 2004; Heilmann et al., 2019). However, rescuing VSV by recombinant vaccinia virus expressing the T7 RNA polymerase (vTF-7.3) is still commonly used in many labs since it is significantly more efficient than other systems. Moreover, as a critical rescue factor, almost all of the reports just showed the MOI range (5–20) of vTF-7.3 (Ostertag et al., 2007; Tani et al., 2011). Determining the appropriate viral load for rescue efficiency and cell death is necessary, which makes some research groups uncertain.

In this study, various MOIs of vTF-7.3 were screened for rescuing recombinant VSV expressing mCherry red fluorescent protein (rVSVmc-p) to measure the rescue efficiency in the further experiments. To avoid producing defective viral particles and the possible vTF-7.3 plaque in BHK-21 cells, the red fluorescence plaques were exclusively counted under fluorescence microscopy, which only allows the observation of the specific focus formation. It has been noted that MOI of five generated viral plaques by 5.2 × 10^3^ PFU/ml, which was further corroborated by qPCR and flow cytometry, which showed a reduction of MOI of one in the genome copies. These would indicate that more viral particles were produced during infection. Furthermore, Western blot also showed comparable results, in comparison to qPCR, which revealed that the group with MOI of five had a considerably larger proportion of infectious viral particles than the group with MOI of 1. Altogether, when all factors are considered, an MOI of five is ideal for rescuing VSV, whereas MOIs of 10 and 20 are not.

vTF-7.3 has been used to construct the reverse genetic systems for many viruses, including the Sendai virus, mumps virus, and hepatitis A virus (Pelet et al., 1996; Bousse et al., 2002; Kusov et al., 2002; Ammayappan et al., 2016). Although this approach has certain disadvantages, such as the necessity for plaque purification, it is still the most widely utilized to rescue viruses since it has the highest rescue effectiveness. We previously experienced a severe problem where BRS-T7 cells perished throughout the infection with unpurified vTF-7.3. It took us a half year to determine that purifying vTF-7.3 is critical to solving it. To verify that, an MTT assay was performed and showed that the unpurified vTF-7.3 has considerable cytotoxicity for BSR-T7 cells, indicating it is a pivotal point in the rescue of VSV.

To sum up, our study demonstrated that the efficiency of rescue VSV is closely related to the various MOI of vTF-7.3 and was purified first. It might help clear up some of the ambiguity surrounding the development of the VSV reverse genetic system.

## Data Availability Statement

The original contributions presented in the study are included in the article/supplementary material, further inquiries can be directed to the corresponding author.

## Author Contributions

WL and FY designed the experiments. FY, JT, YF, YZ, and WL carried out the experiments. The data were analyzed by FY, QH, WH, YL, GC, BF, ZJ, and WL. FY and WL wrote the manuscript. All authors contributed to the article and approved the submitted version.

## Conflict of Interest

The authors declare that the research was conducted in the absence of any commercial or financial relationships that could be construed as a potential conflict of interest.

## Publisher’s Note

All claims expressed in this article are solely those of the authors and do not necessarily represent those of their affiliated organizations, or those of the publisher, the editors and the reviewers. Any product that may be evaluated in this article, or claim that may be made by its manufacturer, is not guaranteed or endorsed by the publisher.
